# The neurobiology of aesthetic chills: How bodily sensations shape emotional experiences

**DOI:** 10.3758/s13415-024-01168-x

**Published:** 2024-02-21

**Authors:** Felix Schoeller, Abhinandan Jain, Diego A. Pizzagalli, Nicco Reggente

**Affiliations:** 1 Institute for Advanced Consciousness Studies, Santa Monica, CA USA; 2 Media Lab, Massachusetts Institute of Technology, Cambridge, MA USA; 3 McLean Hospital, Harvard Medical School, Belmont, MA USA

**Keywords:** Chills, Dopamine, Precision, Reward, Learning, Music, Film, Emotional, Valence, Arousal

## Abstract

The phenomenon of aesthetic chills—shivers and goosebumps associated with either rewarding or threatening stimuli—offers a unique window into the brain basis of conscious reward because of their universal nature and simultaneous subjective and physical counterparts. Elucidating the neural mechanisms underlying aesthetic chills can reveal fundamental insights about emotion, consciousness, and the embodied mind. What is the precise timing and mechanism of bodily feedback in emotional experience? How are conscious feelings and motivations generated from interoceptive predictions? What is the role of uncertainty and precision signaling in shaping emotions? How does the brain distinguish and balance processing of rewards versus threats? We review neuroimaging evidence and highlight key questions for understanding how bodily sensations shape conscious feelings. This research stands to advance models of brain-body interactions shaping affect and may lead to novel nonpharmacological interventions for disorders of motivation and pleasure.

## Introduction

Aesthetic chills (AC) are a strong emotional response to specific stimuli (chills stimuli [CS]), such as music, films, or speech (Schoeller et al., 2022, 2023a, b, c, d), with a characteristic neural signature involving the mesocortical “reward” pathway (Blood & Zatorre, 2001; Ferreri et al., 2019; Salimpoor et al., 2011). Crucially, AC is associated with strong, discrete physiological response of shivering and/or goosebumps (Benedek & Kaernbach, 2011), which ordinarily regulate body temperature (hence AC are perhaps best characterized as “psychogenic shivers”), thereby providing a unique opportunity to study how brain and body interact during consummatory pleasure and reward learning (Contreras-Huerta et al., 2023; Paulus & Stewart, 2014). Interestingly, there seems to be two symmetrical types of AC: 1) positive chills, tied to high rewards (Blood & Zatorre, 2001), and 2) negative chills, associated with high risks (Zald & Pardo, 2002). Both activate the extended amygdala (AMG) and are connected to the processing of uncertainty, whether unexpectedly higher or lower than anticipated (Schoeller & Perlovsky, 2016). The evidence that AC engage both appetitive and aversive neural systems is a reminder of philosophical discussions concerning the nature of awe and the sublime as a mixture of positive and negative emotions (Burke, 1757; Kant, 1951; Schiller; 1967; Longinus, 2022) and work on threat and reward processing in the brain (Murty et al., 2023). The dynamics of AC offer a distinctive window into the emergence of conscious feelings—e.g., how the latency between an *unexpected* interoceptive signal (i.e., shivers and downstream effects, such as goosebumps) and conscious awareness impacts the valuation of exteroceptive cues (Craig, 2008; Damasio & Damasio, 2023; Seth, 2013). Hence, in addition to providing novel insights into how pleasure comes about in the mind and brain, an understanding of the neural process supporting AC could lead to nonpharmacological substitutes for dopaminergic-related illnesses, as suggested by the recent findings suggesting that CS seem to improve reward learning (Jain et al., 2023a, b) and maladaptive cognitions in anhedonic depression (Schoeller et al., 2023a, b, c, d). CS include music (Blood & Zatorre, 2001; de Fleurian & Pearce, 2021), films (Schoeller et al., 2022; Schoeller, Eskinazi et al., 2018a; Schoeller & Perlovsky, 2016), stories (Schoeller & Perlovsky, 2015), poetry or speech (Wassiliwizky et al., 2017; Wassiliwizky & Menninghaus, 2021), scientific practice (Schoeller, 2015a), and religious and secular rituals (Schoeller, 2015a). In this short perspective, we review the neural mechanisms of chills and reward (Section "Neural mechanisms of aesthetic chills and reward") and relate them to the notion of precision encoding in the framework of predictive coding (section "Dopamine and precision encoding in aesthetic chills"). We discuss the notion that chills may be related to the overall predictability of events given previous expectations (a.k.a., precision encoding) and conclude by exploring how AC could influence dysfunctional precision-weighting in psychopathology.

## Neural mechanisms of aesthetic chills and reward

**Fig. 1:**
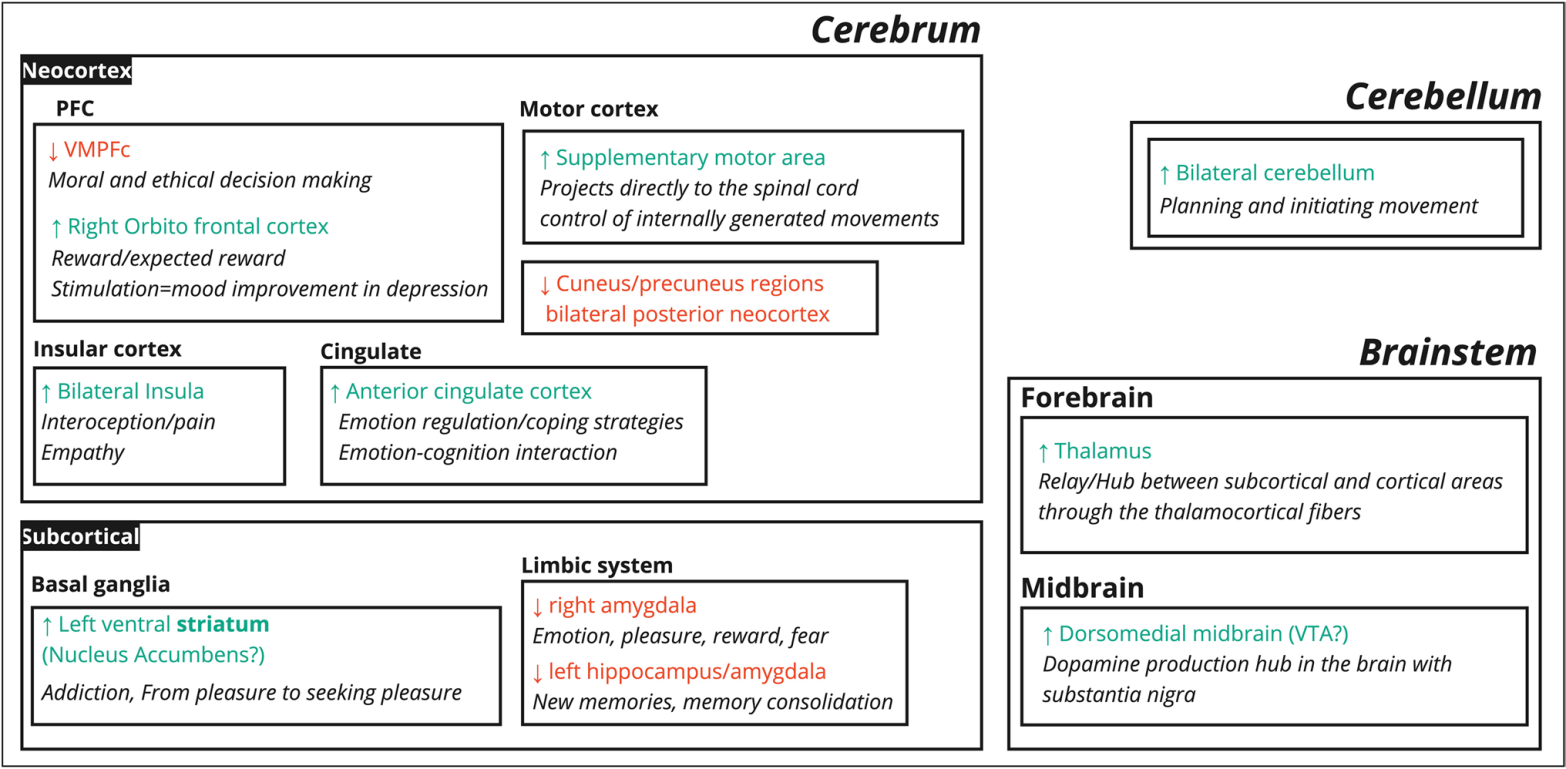
Neural mechanisms of chills and reward. Green areas indicate increased activation during the chills response. Red areas are deactivated. Key regions involved include the ventral tegmental area (VTA) projecting to the ventral striatum (nucleus accumbens) and the hippocampus. The amygdala, orbital, and ventromedial prefrontal cortex show deactivation during chills (Blood & Zatorre, 2001). The VTA, nucleus accumbens, and striatum are part of the brain's reward system, associated with pleasure, reward, and compulsive behavior. The right orbitofrontal cortex (OFC) plays a role in sensory processing, reward, and expected outcomes. Note that electroencephalographic recording of chills showed theta activity with activation in the orbitofrontal cortex, in correlation with emotional ratings (Chabin et al., 2020)

Evidence to date suggests that AC engage a distinct brain network where neurons in the ventral tegmental area (VTA) of the midbrain disperse dopaminergic projections throughout the mesocorticolimbic system to exert neuromodulatory actions critical for a range of reward and motivation processes, including hedonics, reward-related learning, and behavioral adaptation (Blood & Zatorre, 2001; Chabin et al., 2020; Ferreri et al., 2021; Witt et al., 2023; Salimpoor et al., 2011; Zald & Pardo, 2002). As we discuss in the following section, dopamine is thought to encode precision—statistically, the inverse of variance—of predictions, signaling when neural representations are more reliable (Friston et al., 2012)—although see alternative interpretations in Jeong et al. (2022). The relevant circuits are usually broadly characterized as the salience and reward networks, and as such include limbic (amygdala [AMG] and nucleus accumbens [NAcc]) and frontal (orbitofrontal [OFC] and ventromedial frontal cortex [vmPFC]) regions (Seeley, 2019; Sesack & Grace, 2010). As of this article, only seven studies have examined the neural correlates of AC (Blood & Zatorre, 2001; Chabin et al., 2020; Ferreri et al., 2019; Witt et al., 2023; Sachs et al., 2016; Salimpoor et al., 2011; Zald & Pardo, 2002). Unfortunately, the neuroimaging work conducted so far has focused exclusively on music as an eliciting stimulus (but see Wassiliwizky et al., 2017). We simply cannot conclude anything yet regarding AC induced by CS, such as films, speeches, stories, science, and rituals at large. A recently validated, “gold standard” database of CS, extending beyond music, has been established by Schoeller et al. (2022) later validated on 3000+ participants (Schoeller et al., 2023a, b, c, d), marking the initial steps in uncovering the underlying neural mechanisms beyond mere musical pleasure. We review some of the neural correlates associated with musical chills as a springboard (Fig. 1), with all the limitations entailed.

Blood & Zatorre (2001) used PET imaging to investigate musical chills, reporting increased blood flow to the ventral striatum, midbrain, insula, right orbitofrontal cortex, thalamus, anterior cingulate, SMA, and cerebellum. The decrease in blood flow was noted in areas, such as the amygdala, left hippocampus, and vmPFC. The authors observed that this profile is similar to neural responses in cocaine-dependent subjects when administered cocaine (Breiter et al., 1997), suggesting that musical chills may activate pathways akin to those engaged by euphorogenic substances (Biederman & Vessel, 2006; Nguyen et al., 2021). Given the causal relationship between dopamine and the pleasure derived from cocaine (Volkow et al., 1999), it raises the question of whether the sensation of chills may engage a dopaminergic response analogous to that which is classically observed during “incentive salience” (Berridge, 2007). Early studies by Goldstein suggest that chills can be inhibited by an opioid-antagonist (Goldstein, 1980), but these results have failed to replicate (Laeng et al., 2021). In the study by Laeng et al. (2021), compared with placebo, the opioid antagonist (50 mg, naltrexone) did not alter self-reported pleasure associated to the CS, but it did specifically reduce pupil size during AC. This suggests that while the endogenous μ-opioid system is not essential for the subjective pleasure of AC, blocking opioids induce a reduction in arousal in response to the CS.

The influence of dopamine on various functions—such as sensory pleasure (also known as hedonic impact), increased motivation, and learning—is evident in overlapping brain regions, including the ventral striatum (NAcc), midbrain (VTA, Periaqueductal Gray [PAG], Pedunculopontine Nucleus), amygdala, hippocampus, mPFC (Berridge & Robinson, 1998; Terry et al., 1995). Consistent with this line of reasoning, Salimpoor et al. (2011, 2013) subsequently used PET to demonstrate that the pleasure derived from music is associated with dopamine release in the dorsal and ventral striatum, specifically the NAcc. This neural activity was found to correlate with how much participants would pay for pleasurable songs, connecting the experience of musical pleasure with reward-based decision making. Furthering confidence in this relationship, Mas-Herrero et al. (2018) demonstrated a causal link between neural activity and music appreciation, where excitatory TMS to the left DLPFC increased pleasure, arousal, and monetary valuation of music (i.e., the subject’s willingness to spend money on songs), while inhibitory TMS had the opposite effect. Ferreri et al. (2019) also showed that the administration of dopamine precursors, such as levodopa upregulates the experience of pleasure during music listening (but not valence and arousal), and drives a significant increase in chills incidence, whereas dopamine antagonists reduced these effects compared with placebo (Ferreri et al., 2019).

These results suggest that aesthetic pleasure may be rooted in the interplay between reward/valuation systems (striatal-limbic-paralimbic) and more phylogenetically advanced perception/prediction systems (temporofrontal). Goupil & Aucouturier (2019) proposed models that highlight two distinct, yet possibly, interacting frameworks: (A) a corticostriatal model connected with NAcc for musical pleasures, and (B) a model for musical emotion involving the thalamus, amygdala, and DLPFC. The orchestration of these responses raises profound questions about whether A and B operate independently, if A provides first-order input to the construction of emotion, or if A evaluates B, thereby illuminating the complex neural interactions that underpin the experience of pleasure and emotion.

**Fig. 2:**
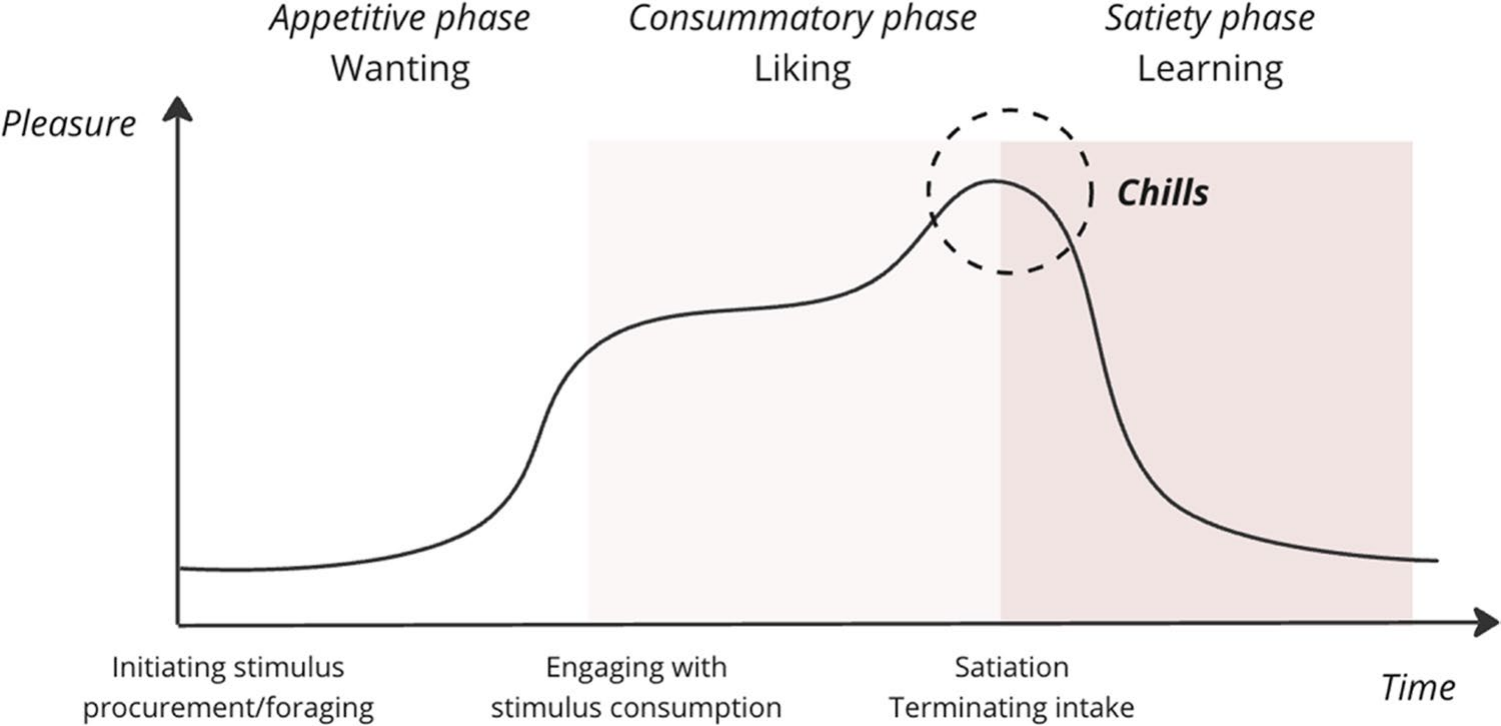
Three phases of pleasure and chills as peak consummatory pleasure. The "Wanting" phase represents the initial anticipation and desire for rewards. The "Liking" phase corresponds to the peak of consummatory pleasure, characterized by the experience of aesthetic chills (AC). Following the chills, the “Learning” phase begins, involving the encoding of crucial information about the film. This phase is associated with the consolidation of the CS meaning. In other words, AC marks the onset of the satiation process, where the viewer's curiosity is temporarily satisfied

Interestingly, the brain correlates of chills map with the first phase of the reward cycle (a.k.a., the “Wanting” phase), characterized by midbrain dopamine projections to forebrain targets, such as NAcc and other parts of striatum (Berridge et al., 2009) (Fig. 1). This contrasts with the dynamics of AC in narrative films, where AC generally arise during the culmination of the film—not beforehand (Fig. 2). In other words, people do not experience chills in anticipation of the movie; rather, these sensations tend to manifest during the film itself (although an exception could be made of film “trailers” as evidenced in Schoeller et al., 2023a, b, c, d). This is when the narrative's uncertainties are resolved, and a cohesive meaning emerges from the tapestry of events that unfold throughout the film—i.e., the film is over (Schoeller & Perlovsky, 2015). This pattern would instead suggest that AC correspond to a peak in consummatory pleasure (a.k.a., the “Liking” phase), kicking off the satiation process (a.k.a., the “Learning” phase) (Schoeller & Perlovsky, 2016), where mesolimbic circuitry is responsible for generating incentive salience (Berridge, 2012). Note that the desire for rewards also is involved in generating fearful salience (Berridge & Kringelbach, 2015), consistently with previous research on negative AC by Zald & Pardo and the “reversed role” of the amygdala in positive and negative AC during aversive auditory stimulation (Zald & Pardo, 2002). To reconcile this, we consider the neurobiological underpinnings of pleasure and satisfaction, acknowledging that the neural circuits involved—namely the Nucleus Accumbens (NAc), Ventral Tegmental Area (VTA), and striatum—do not necessarily exhibit exclusive activity in distinct phases of appetitive, consummatory, and satiety processes. Instead, these regions play a pivotal role in transitioning from consumption to satiety, as evidenced by studies showing NAc’s role in initiating and maintaining feeding behaviors, and its potential 'unpausing' during the transition to satiety (Krause et al., 2010). Furthermore, VTA lesions have been associated with reduced overconsumption (Shimura et al., 2002), suggesting its involvement in peak consumption and the subsequent shift to satiety. This is further supported by evidence of dopamine release in the striatum during peak consummatory pleasure (Small et al., 2003). Therefore, we propose that AC may not solely be an artifact of the “wanting” phase but also could represent a neurobiological marker of the transition from consumption to satiety, characterized by a relative increase in neural activity (e.g., from pause to unpause in NAc activity). This perspective aligns with the observed overlap in neural correlates and the temporal dynamics of AC. Additionally, we acknowledge that the temporal resolution of fMRI may not be sufficiently sensitive to capture these transient processes, especially considering the challenges in perfectly timing the self-reporting of chills with neural activity.

**Fig. 3:**
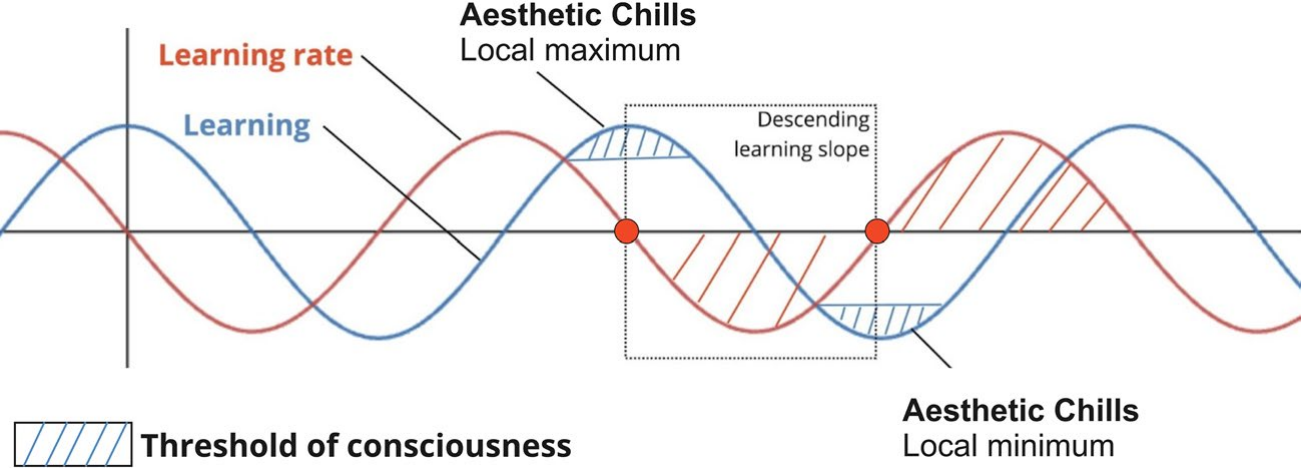
Learning (blue) and its rate of change (red), as the fit between ascending signals and available models. Learning is represented by the equation: L = ∏n [∑m l(X(n) | M(m))], where l denotes the conditional similarity of data given conditional models (Schoeller et al., 2018a, b). This sinusoidal representation is a gross oversimplification for the sake of readability. The blue area at the peak of the curve defines conscious aesthetic emotions (when the rate of change tends toward zero), and the squared area describes a descending learning slope corresponding to a negative derivative. The graph highlights the interplay between learning and emotions, emphasizing how insights (e.g., positive chills) and traumas (e.g., negative chills) shape an individual’s overall mood and well-being. Adapted from Schoeller & Perlovsky, 2016

Schoeller & Perlovksy suggested a computational models for AC (Schoeller & Perlovsky, 2016) as a peak value in uncertainty reduction at higher levels of cortical information encoding—represented by the equation: L = ∏n [∑m l(X(n) | M(m))], where *l* denotes the conditional similarity of sensory signals given available models (see technical details in Schoeller Perlovsky, et al., 2018b). Importantly, the local peak value (i.e., modelled as a null derivative) is independent of valence, accounting for the fact that chills can be experienced in response to both positive (appetitive) and negative (aversive) stimuli (Fig. 3). In line with recent models of affect as second order predictions about learning, i.e., expected prediction errors (Joffily & Coricelli, 2013; Kenett et al., 2023; Miller & Clark, 2018; Perlovsky & Schoeller, 2019; Sarasso et al., 2020; Van de Cruys & Bervoets, 2022), this suggests that chills may correspond to a null learning rate, or zero derivatives, signaling a local maximum (extremum) in learning (Schoeller, Perlovsky et al., 2018b; Schoeller & Perlovsky, 2016). In other words, AC occur when the individual reaches the limits of their ability to learn more about their surroundings, and their learning rate slows down, terminating the Wanting phase and kicking off the Satiation phase, effectively corresponding to what has been described in the field as a temporary satiation of curiosity (Biederman & Vessel, 2006; Kenett et al., 2023; Sarasso et al., 2020; Schoeller, 2016). In machine learning, momentum-based gradient optimization algorithms utilize a similar concept of momentum to expedite the convergence process toward minima in the error landscape, enhancing the efficiency of learning in areas with consistent error reduction (Ruder, 2016). These methods mirror the adaptive modulation of reward cycles, observed in both biological (Soltani & Koechlin, 2022) and artificial (Piao et al., 2023) intelligent, self-organizing systems. In both cases, the dynamic, adaptive recalibration of reward mechanisms is pivotal for optimizing learning trajectories. The mammalian brain, particularly in the prefrontal cortex, is equipped with computational abilities to form and evaluate task sets based on internal models associating stimuli, actions, and outcomes. This configuration facilitates adaptive behavior through selective, predictive, and contextual models, integrating rewards, statistical inferences, and dynamic environmental cues. Additionally, the activation of motor regions of the cortex, such as the cerebellum or the supplementary motor area, further supports the hypothesis that chills may be related to action orientation and readiness (Sarasso et al., 2019, 2020, 2021, Sarasso, Francesetti et al., 2022a, Sarasso, Neppi-Modona et al., 2022b). Sarasso and colleagues accumulated extensive data on the role of aesthetic emotions in learning (and their downstream effects in terms of perceptual and memory improvement) and suggested the hypothesis that they may be related specifically to the inhibition of action for the purpose of seeking and acquiring knowledge (Sarasso et al., 2019, 2021, Sarasso, Neppi-Modona et al., 2022b), in line with the hypothesis that AC may correspond to a satiation of curiosity (Schoeller, 2015b, 2016).

Activity in the insular cortex during the chills response hints towards the role of interoception (the perception of bodily sensations) and peripheral signals in the emotional experience of chills (Craig, 2008; Damasio, 1996; Seth, 2013). Recent evidence suggests that lesions impairing structural connectivity of the left insula in stroke patients modulated AC (Witt et al., 2023). Specifically, a large left hemispheric lesion including the left insula impaired the bodily response of chill experience (objective chill response) but left the cognitive aspects of chill processing (subjective chill response) unaffected (Grunkina et al., 2017). The study by Witt et al. (2023) examined stroke patients who primarily had damage to the insula. The study found that these patients experienced chills in response to auditory stimuli (such as music or harsh sounds) at a frequency similar to that of people without brain damage. However, there was a disconnect between their emotional arousal and their physical responses that correlated with structural connectivity between the left anterior insula and the temporal pole. Additionally, fMRI analyses revealed that during chills stimuli, reduced skin conductance responses correlated with lower activation of the temporal pole. These findings highlight a role for the insula and temporal pole in integrating physiological responses to arousing experiences like chills. This role of interoceptive signals in chills also is evident in data showing that physical manipulation of the somatic markers of the emotion by means of a wearable prosthesis enhancing the sensation of cold can enhance the feeling of pleasure and some of the downstream effect of chills (Haar et al., 2020; Ishikawa et al., 2023; Jain et al., 2020; Schoeller et al., 2019). Interoceptive signals provide essential information to the brain about the body’s internal state, contributing to the shaping of emotional responses and the anticipation of rewarding outcomes (Contreras-Huerta et al., 2023; Paulus & Stewart, 2014). Positive stimuli, whether they are social interactions, delicious food, or soothing experiences, such as the warmth of sunlight, activate neural pathways that are closely intertwined with interoception. Conversely, negative experiences or threats can trigger aversive responses that also involve interoceptive processing, contributing to our understanding of interoception's role in the broader spectrum of emotions. Notably, amygdala and insula connectivity modulate the effect of input arousal on effective connections, with the amygdala as the main hub driving arousal input (Wang et al., 2023).

Elaborating on the contributions of insular-cortical connectivity to the uptake of arousing stimuli, Sachs et al. (2016) accounted for individual differences in the trait frequency of experiencing musical chills with white-matter tract volume (an indicator of structural connectedness (Mollin et al., 2016; Seehaus et al., 2013, 2015)) within a three-node network of the posterior superior temporal gyrus (auditory association area) with the anterior insula (interoception and emotion processing (Zaki et al., 2012)) and medial prefrontal cortex (reward processing (Knutson et al., 2003; Tzschentke, 2000; Pastor and Medina, 2021)). These findings, which illustrate that increased sensory access (i.e., increased structural connectedness) to reward systems accounts for increased sensitivity to chills (Mori & Iwanaga, 2015), suggest a potential evolutionary foundation for the aesthetically rewarding function of meaning-making in humans. Given the evidence that practice induces microstructural white-matter changes across several domains and time scales (Sagi et al., 2012; Engvig et al., 2011; Reid et al., 2017), it stands to reason that these effects could be interpreted in reverse: frequent chills might lead to enhanced structural connectivity between these regions. Future research should investigate the structural effects of repeated exposure to chills to better understand any causal relationships involved.

Individual differences in the propensity to experience aesthetic chills also have been linked to both personality traits, such as absorption and openness to experience (Silvia & Nusbaum, 2011, Williams et al., 2018, Williams et al., 2023, Silvia et al., 2015, Johnson et al., 2023, Schoeller et al., 2023a; McCrae, 2007), as well as biological factors, such as gene polymorphisms affecting neurotransmitter function. A recent twin study found that approximately 36% of variance in aesthetic chills experiences can be attributed to genetic factors (Bignardi et al., 2022). This further supports a role for underlying biological pathways in facilitating intense emotional responses to aesthetic stimuli. Given evidence that serotonin and dopamine systems interact, with dopamine D2 and serotonin 5-HT1a receptors showing functional opposition (Howell & Cunningham, 2015), an interesting question is whether altering serotonin function could inhibit AC by shifting this balance. For example, increased serotonin signaling through selective serotonin reuptake inhibitors (SSRI) antidepressants might conceivably dampen chill experiences mediated by dopamine release. Testing effects of pharmacological manipulations on aesthetic chills could elucidate neuromodulatory mechanisms underlying individual differences in emotional reactivity.

## Dopamine and precision encoding in aesthetic chills

Within a view of the brain as an organ of prediction and learning (Kveraga et al., 2007; Downing, 2009), these findings and our knowledge of the brain structures marry well with the growing amount of evidence that dopaminergic neurons and pathways play an essential role in learning by encoding the precision of prediction errors (i.e., their confidence or reliability) along the cortical hierarchy (Diederen et al., 2017; Jeong et al., 2022). What’s more, the peculiarity of the chills phenomenon make it an excellent object of experimental study, insofar as chills can be 1) consciously reported by subjects, 2) are, in principle, measurable physically in terms of heat dispersal, and 3) seem universal across cultures (McCrae, 2007). Precision encoding is a process by which the brain continuously estimates the reliability of the sensory inputs (i.e., the prediction errors) encountered and the reliability of its models of the world (Keshvari et al., 2012). Crucially, mounting evidence indicates that dopaminergic pathways mediate this process, where dopamine release occurs when reward is greater than expected, which helps the brain update predictions and improve learning trajectories (Schultz, 2016; Kiverstein et al., 2017). Precision encoding may be related to the enhancement of memory and attention processes observed during AC experiences (Ferreri et al., 2019, 2021). Recent evidence by Kathios et al. shows that when people are exposed to novel musical melodies based on an unfamiliar scale, liking ratings increase for frequently presented melodies adhering to an implicitly learned structure, supporting predictive coding models whereby new musical sounds become rewarding through learned predictions (Kathios et al., 2023). By encoding the precision of prediction errors, dopamine allows the brain to consolidate and retain information, leading to improved memory and attention of salient events (Adcock et al., 2006; Ferreri et al., 2019, 2021; Sarasso et al., 2021).

In a series of experiments, it was repeatedly shown that priming subjects with incoherent stimuli inhibits the chills (Schoeller & Perlovsky, 2016; Schoeller & Eskinazi, 2019; Schoeller et al., 2018b), most likely as incoherence (i.e., surprisal) interferes with the precision system (Schoeller & Perlovsky, 2016). CS activate dopamine projections, aiding to the formation and consolidation of emotional memory (Ripolles et al., 2016; Ferreri & Rodriguez-Fornells, 2017, 2022; Ferreri et al., 2021, 2013; Sarasso et al., 2019, 2020, 2021, Sarasso, Francesetti et al., 2022a, Sarasso, Neppi-Modona et al., 2022b). Sarasso and colleagues accumulated evidence that chills and aesthetic emotions induce an enhancement of memory and attention processes, corroborating their hypothesis and confirming previous studies by Laura Ferreri on reward-driven music memory consolidation and the role of dopamine in the process (Ferreri & Rodriguez-Fornells, 2017, 2022; Ferreri et al., 2021, 2013). Neurobiologically, this makes sense insofar as VTA dopaminergic projections to the hippocampus, as observed in the chills phenomenon, are known to play a crucial role in encoding emotional memories (Ripolles et al., 2016; Shohamy & Adcock, 2010). The extended amygdala’s involvement calls for studies on the roles of acetylcholine and its combined effects with dopamine in supporting aesthetic chills and their connection to learning. That is because acetylcholine, acting through nicotinic receptors, interacts with dopamine release and may contribute to precision signaling (Matityahu et al., 2023). Along similar lines, musical pleasure stems from expectations generated by learned musical patterns and the rewarding violations of those expectations (Krumhansl & Agres, 2008; Koelsch et al., 2019, Kraus, 2020).

As we engage with CS, our brain continually predicts upcoming events based on implicit knowledge (Zatorre & Salimpoor, 2013). Dopamine signals violations of expectations, or prediction errors, driving learning to update expectations (Pessiglione et al., 2006; Egermann et al., 2013). High precision tunes attention—enhancing memory and learning—and the sudden drop in precision induced by an incoherent prime interrupts this process (Schoeller & Perlovsky, 2016; Schoeller & Eskinazi, 2019; Schoeller et al., 2018b). Precision tuning focuses attention and learning on the most reliable predictions. Musical or narrative tension builds uncertain predictions, engaging a cascade of stimulus-driven expectations until resolution ultimately satisfies the predictions, eliciting pleasure (Lehne et al., 2014, Schoeller & Perlovsky, 2016—see also Deterding et al., 2022 in the context of video games). Kathios et al. (2023) demonstrated that functional MRI activity in auditory cortical regions reflected prediction errors, while connectivity between auditory and medial prefrontal cortex tracked both exposure and prediction error signals, further implicating predictive coding in the context of musical reward. Across nine studies (n = 1,185), statistical learning of sequential patterns was sufficient to drive rewarding responses to expectation violations for novel musical systems (Kathios et al., 2023).

CS engage a cyclic interplay between cortical systems generating expectations, dopamine signaling of prediction precision and errors to update expectations, and subcortical systems mediating emotion, reward, and memory. The arousal system plays a significant role in this process, including dopaminergic pathways, regulating sensory processing and emotional responses (Pfaff & Banavar, 2007). Elevations in dopamine release within mesolimbic, mesocortical, and nigrostriatal target sites coincide with arousal (Horvitz, 2000). Notably, the level of arousal before CS exposure is a major predictor of whether the subject will experience chills subsequently (Schoeller et al., 2023b); chills participants report arousal levels twice as high as nonchills participants on average before the CS exposure. This aligns with theories proposing primal brain areas as central neural correlates of consciousness (Solms, 2021; Safron, 2021; Schoeller et al., 2023a, b, c, d). AC and aesthetic emotions may initially originate from basic autonomic emotional responses rather than higher-order cognitive processes, which would explain why chills may feel surprising when they finally enter consciousness.

**Fig. 4:**
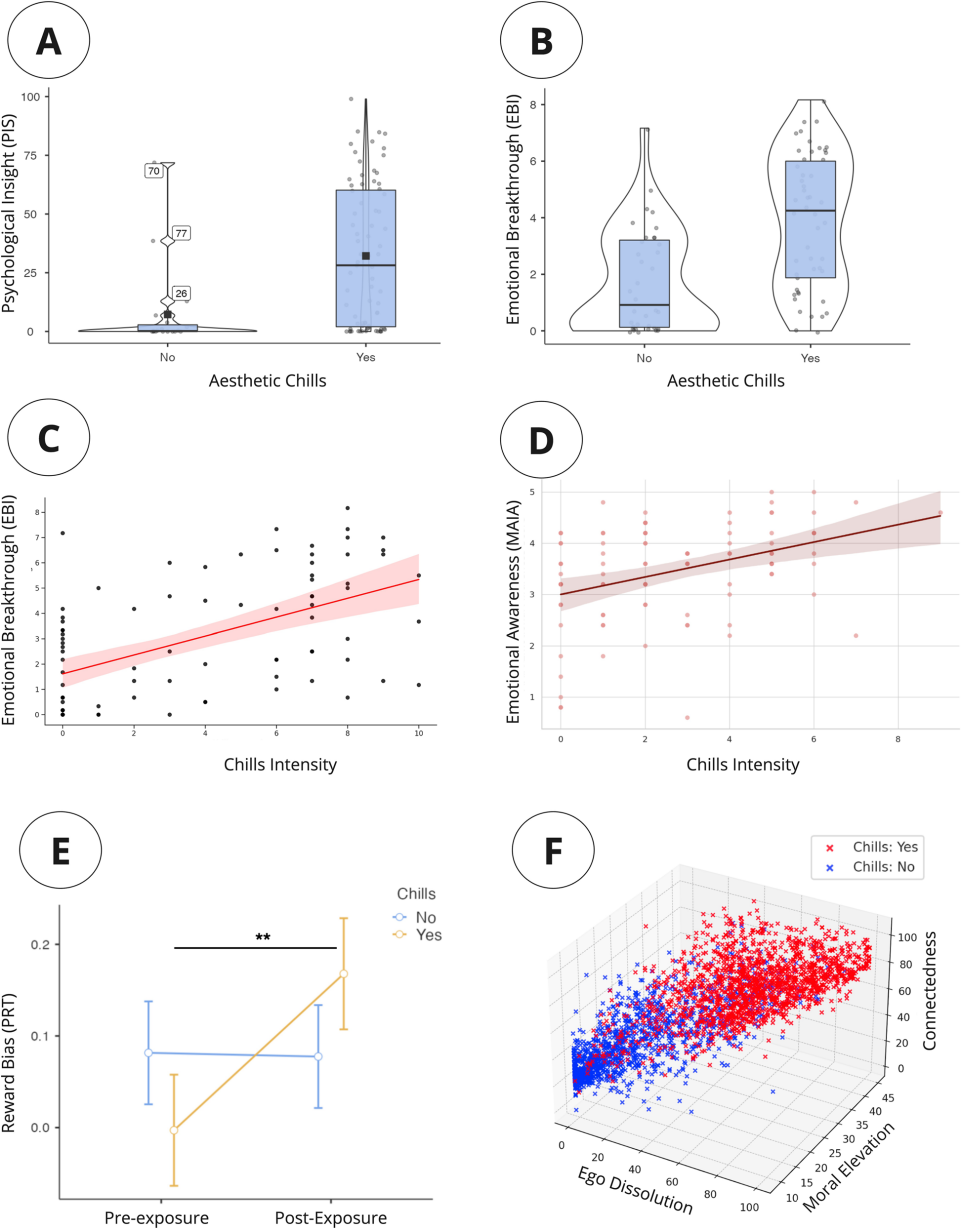
**A** and **B.** Individuals who experience aesthetic chills display higher psychological insight (PIS) and emotional breakthrough (EBI) compared with control (Schoeller et al., 2023c; 2023d). **C** and **D.** Chills intensity is positively correlated with emotional breakthrough (EBI) and emotional awareness (MAIA). **E.** AC was associated with a significant change in reward bias (PRT) pre- and post-exposure to stimuli in subjects compared to control (Jain et al., 2023b). **F.** Experience of aesthetic chills was reliably associated with patterns of ego dissolution, connectedness, and moral elevation (Christov-Moore et al., 2023)

Notably, dysfunctional precision encoding of prediction errors by dopamine is implicated across psychiatric illnesses, including schizophrenia, depression, and addiction. For example, in schizophrenia, the precision of perceptual priors driving inference is pathologically low, leading to hallucinations and delusions. Dopamine has been suggested to play a role in the precision weighting of unsigned prediction errors, which signal the degree of surprise without indicating valence (Haarsma et al., 2021). Hence, dysfunctional precision-weighting of unsigned prediction errors may be a pivotal contributor to the pathogenesis of psychosis (Krystal et al., 2017; Adams et al., 2013; Heinz et al., 2019). In contrast, high precision in depression (especially concerning social cues) may reflect impaired reward sensitivity and learned helplessness (Smith et al., 2023). Hence, identifying the relationship between aesthetic chills, the precision-weighting of prediction errors, and its dopaminergic substrate could help to understand the role of dopamine in these conditions. Using a recently constituted database of CS, some preliminary studies have investigated the effects of aesthetic chills on subjects diagnosed with Major Depressive Disorder (Jain et al., 2023a; Schoeller et al., 2023c). Preliminary data suggest that chills have an effect in mood disorders (Jain et al., 2023a, b; Schoeller et al., 2023c). A first study (Jain et al., 2023b) investigated whether experiencing aesthetic chills could improve reward learning in 103 people with depressive symptoms, especially anhedonia, using videos known to elicit chills and the Probabilistic Reward Task (PRT) (Pizzagalli et al., 2008). Anhedonic participants who experienced chills showed a significant increase in reward bias on the PRT compared with those who did not experience chills (*p* = 0.004), suggesting temporary mitigation of blunted reward learning; however, no difference was seen in non-anhedonic depressed participants (Fig. 4E).

Another study on depression investigated whether experiencing aesthetic chills could shift maladaptive self-beliefs (Schoeller et al., 2023c). The findings revealed that chills positively alter (depressogenic) negative self-schemas (e.g., related to shame and self-acceptance) (Schoeller et al., 2023c). Crucially, the phenomenology associated to the belief change induced by CS closely resemble the effects observed in psychedelic-assisted therapy, including the characteristic associated phenomenology of emotional breakthrough (EBI) (Roseman et al., 2019; Schoeller et al., 2023c), psychological insight (PIS) (Peill et al., 2022; Schoeller et al., 2023d), and ego dissolution (Nour et al., 2016; Christov-Moore et al., 2023) (Fig. 4). The surprising, involuntary nature of chills, their emergence without conscious learning, and their underlying neurobiology suggest a strong similarity to so-called primary states of consciousness (Schoeller et al., 2023c), which are considered phylogenetically older states of consciousness often associated with profound changes in beliefs, perceptions, and behavior (Schoeller, 2016; Carhart-Harris et al., 2014). Psychedelic experiences, such as those induced by psilocybin, which stimulates 5-HT2a receptors, often result in similar transcendent experiences, with shared characteristics to aesthetic chills (Schoeller et al., 2023c, d; Christov-Moore et al., 2023). However, currently no direct evidence links serotonin 5-HT2a receptors to AC.

**Fig. 5:**
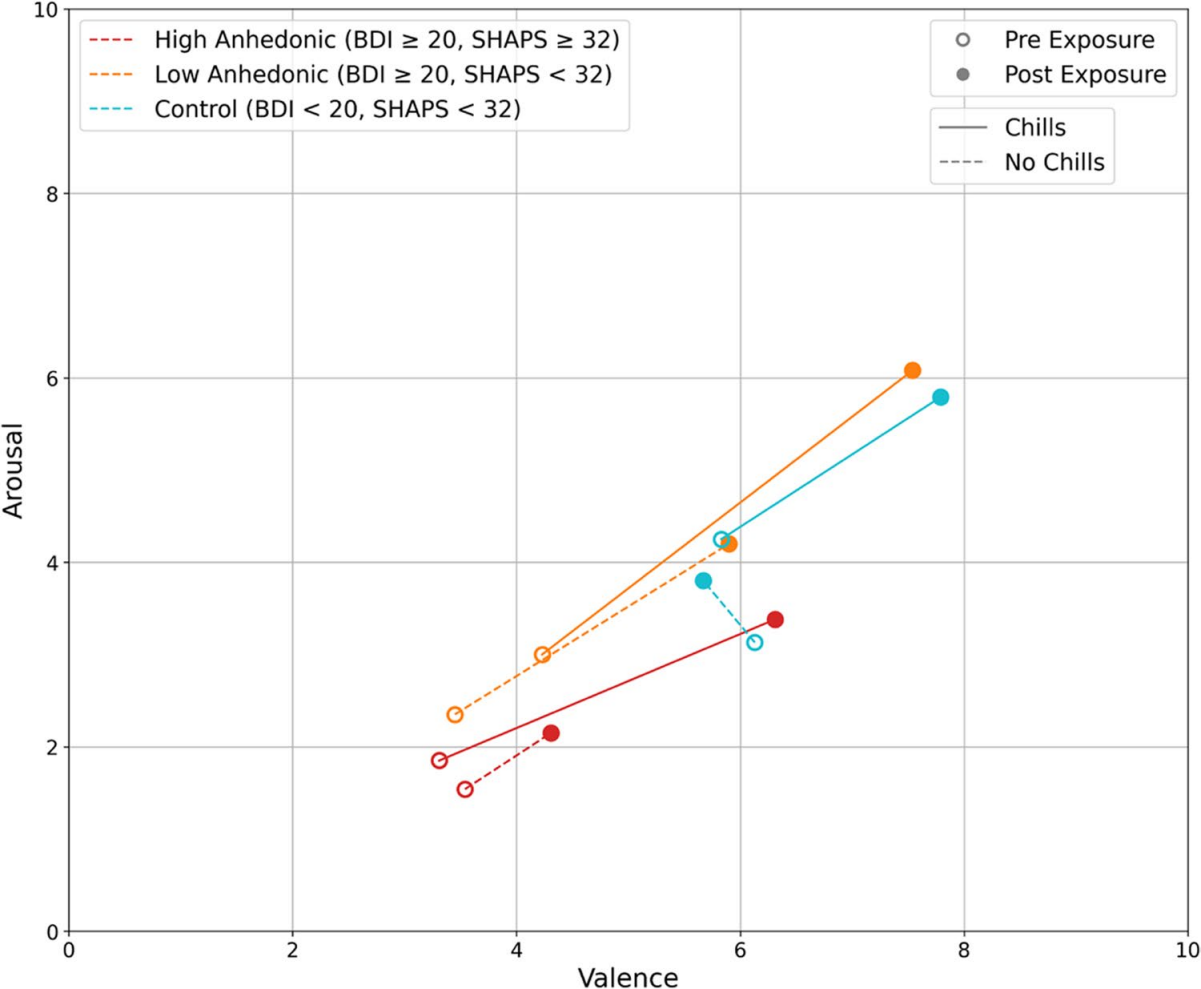
Circumplex model depicts participants’ self-reported arousal and valence before (empty dots) and after chills exposure (full dots). Participants with high anhedonia (HA) are shown in red, low anhedonia (LA) in orange, and healthy controls in blue. Solid lines indicate participants who experienced chills, while dotted lines show those who did not. Empty dots represent pre-exposure ratings, while filled dots show post-exposure ratings. HA participants who experienced chills shifted toward LA participants without chills and control pre-exposure levels. This suggests chills exposure increased arousal and valence in high anhedonia to approach levels seen in low anhedonia and controls

Perhaps the most robust effect found across these studies was AC effects on emotional valence, reliably generating an “emotional drift,” i.e., predictable change in their emotional state, including in participants with anhedonic symptoms (results summarized in Fig. 5). In all of these studies, participants are invited to report their level of emotional valence and arousal before and after the experience (Jain et al., 2023a, b; Schoeller et al., 2022). Participants who experienced chills reported a larger shift in valence scores from pre- to post-assessment across all groups compared with those who did not. Interestingly, in the anhedonic study (Jain et al., 2023b), the endpoint arousal and valence scores for anhedonic patients *after* experiencing chills were almost the same as those for non-anhedonic, depressed individuals, *who did not experience chills*. This suggests that experiencing chills brings the emotional state of anhedonic patients closer to that of depressed patients without anhedonia. Additionally, there was no difference in endpoint affective scores for the depressed group (non-anhedonic) compared with the control group. This indicates that the emotional state of non-anhedonic depressed individuals was similar to that of the healthy control group by the end of the study.

## Conclusions

Aesthetic chills offer a unique opportunity to study reward learning in both healthy and clinical populations because of its universal nature across cultures and its ability to be consciously reported and physically measured. AC activate a specific brain network involving the VTA and its dopaminergic projections to the mesocorticolimbic system, crucial for reward and motivation processes. This network includes NAcc, AMG, and frontal regions, such as the OFC and VMPFC, primarily studied in the context of musical chills. In line with behavioral evidence showing high surprisal inhibits the AC process, dopamine plays a key role in AC, encoding the precision of predictions and enhancing sensory pleasure, motivation, and learning. AC are linked to the reward cycle’s phases: wanting, liking, and learning, representing a peak in consummatory pleasure and the onset of the learning phase. The insular cortex’s role in interoception significantly affects the emotional experience of chills, with individual differences in AC propensity linked to personality traits, structural brain connectivity, and genetic factors. Understanding AC’s neural underpinnings has implications for psychiatric conditions like schizophrenia, depression, and addiction, where dopamine’s role in precision encoding of prediction errors is crucial. Preliminary studies suggest AC can positively affect mood disorders, potentially improving reward learning in anhedonia and altering negative self-beliefs.

## Data Availability

The data is available through the referenced articles.
